# The epidemiology of alcohol utilization during pregnancy: an analysis of the Canadian Maternity Experiences Survey (MES)

**DOI:** 10.1186/1471-2393-11-52

**Published:** 2011-07-12

**Authors:** Meghan J Walker, Ban Al-Sahab, Farah Islam, Hala Tamim

**Affiliations:** 1 Dalla Lana School of Public Health, Division of Epidemiology, University of Toronto, Toronto, ON, Canada; 2 School of Kinesiology and Health Science, York University, Toronto, ON, Canada

**Keywords:** Alcohol utilization, pregnancy, prevalence, predictors, Canada

## Abstract

**Background:**

Maternal alcohol consumption during pregnancy may potentially constitute a major public health concern in Canada but despite this, the available epidemiological data on both rates and predictors of alcohol consumption during pregnancy is limited. The present study assessed the prevalence and predictors of maternal alcohol consumption during pregnancy of women living in Canada from 2005-2006 who had a singleton live birth and whose child remained in their care 5-9 months following birth. Prevalence of maternal alcohol consumption was examined across the Canadian provinces.

**Methods:**

The analysis was based on the Maternity Experience Survey (MES), a population-based survey that assessed pregnancy, delivery and postnatal experiences of mothers and their children between November 2005 and May 2006. The main outcome variable assessed was ever drinking alcohol during pregnancy. The sample of mothers who drank during pregnancy consisted mainly of low to moderate level-alcohol drinkers (95.8%), while only 1.7% of the sample were heavy drinkers (>1 drink per day). Socio-economic factors, demographic factors, maternal characteristics, and pregnancy related factors that proved to be significant at the bivariate level were considered for a logistic regression analysis. Bootstrapping was performed to account for the complex sampling design.

**Results:**

Analysis of 5882 mothers, weighted to represent 72,767 Canadian women, found that 10.8% of women drank alcohol at some point during their pregnancies. This mainly reflects prevalence of low to moderate maternal alcohol consumption. Prevalence of drinking alcohol during pregnancy was 13.8% in Eastern-Central provinces, 7.8% in Western Provinces-British Columbia, 4.1% in Eastern-Atlantic provinces and 4.0% in Western-Prairie Provinces. Utilizing alcohol during gestation was significantly associated with several important factors including marital status, smoking status, reaction to the pregnancy and immigrant status. While being an immigrant to Canada appeared to confer a protective effect, women who have partners (odds ratio (OR) = 2.00; 95% confidence interval (CI): 1.20, 3.31) and smoked during pregnancy (OR = 1.54; 95% CI: 1.12, 1.87) were significantly more likely to drink alcohol during their pregnancies. Perhaps most importantly, pregnant women who reported indifference or being unhappy/very unhappy in regards to their pregnancies exhibited 1.89- and 2.5-fold increased risk of drinking alcohol during their pregnancies, respectively.

**Conclusion:**

A number of important factors associated with maternal alcohol utilization during pregnancy have been identified, indicating areas where increased focus may serve to reduce maternal and pediatric morbidity and mortality.

## Background

Alcohol is a legal, socially acceptable, and frequently abused substance, with the majority of Canadian women of reproductive age reporting its use to varying degrees [1]. The adverse effects on the fetus have been widely studied [2,3]. Reports have been inconsistent regarding the alcohol intake threshold at which such effects occur [3]: some studies report that low-moderate levels of maternal alcohol consumption have no consistent significant effect on birth outcome and fetal malformation [4,5], while other studies indicate even single-dose events can result in observable deficits [1]. Alcohol's teratogenic effects exist along a continuum, ranging from subtle to the most serious outcome, a diagnosis of Fetal Alcohol Syndrome (FAS) [6]. The World Health Organization recognizes the risk of prenatal alcohol exposure and its association with developmental and intellectual disability [7]. It is associated with increased rates of preterm birth and fetal death [8], reduced brain mass [6] and prenatal and postnatal growth retardation [9]. Cognitive deficits have also been demonstrated in language, visuospatial function, fine and gross motor ability, attention, memory and judgment [2,9,10]. Exposure to alcohol in-utero has also been associated with mental health disorders, cardiac, skeletal, renal, ocular and auditory deficits [2,10]. Additionally, the burden in Canada is profound, with adjusted average annual costs per child afflicted with FAS and Fetal Alcohol Effects (FAE) of $14,342 and prevalence rates approximated at 1 to 6 in 1000 live births [11].

While the harmful effects of heavy maternal alcohol consumption and binge drinking, such as FAS and disruption to fetal organ formation, have been demonstrated [12-15], evidence supporting the adverse effects of low to moderate maternal drinking remains inconclusive. Maternal alcohol consumption often occurs in conjunction with other risk factors (eg. smoking and family history of alcohol abuse), and so it is difficult to attribute the effects to fetal alcohol exposure or to the characteristics of the mother and the child's home environment [16]. No evidence has been found to conclusively link low to moderate maternal alcohol consumption with Autism Spectrum Disorder nor infantile autism [17], or longitudinally measured fetal growth characteristics [18], and no evidence has been found correlating light drinking with childhood behavioural difficulties or cognitive deficits [12]. Research has even supported the modest protective effect of light maternal alcohol consumption on restriction of fetal growth during gestation, preterm birth [5], and childhood behavioral difficulties or cognitive deficits [12] compared to mothers who abstained from maternal alcohol consumption.

There is a lack of clear and consistent research supporting the adverse effects of maternal alcohol consumption across all drinking levels. The inconclusive nature of the body of research does not allow for the establishment of a non-harmful threshold for maternal alcohol consumption, and therefore, the public health promotion of no alcohol use during pregnancy is the safest measure to reduce fetal harm. Regardless, many women continue to consume alcohol to various degrees throughout their pregnancies. This is a problem in much, if not all, of the world [19]. It appears from the published data on women's gestational consumption of alcohol that Canadian and U.S. rates have generally been declining since the 1990's. However, Health Canada reports that approximately 15% of pregnant Canadian women still appear to be using alcohol to some extent [20]. An Alberta study found that while 50% of women reported alcohol consumption pre-pregnancy recognition, 18% continued to drink even after post-pregnancy recognition [21]. A review of the Canadian literature, while somewhat lacking, supports this and overall, Canadian rates of maternal alcohol consumption during pregnancy are comparable with those of the U.S., estimated between 5-15% [19,22-25]. Worldwide rates are variable, with other nations including Australia and many European countries reporting higher prevalence [6,8,26]. In one European study, only 53% of women in France reported complete abstinence during pregnancy [8] and an Australian study reported 81% of pregnant women had consumed alcohol [6].

Women consuming alcohol during gestation are far from homogenous, and in order to adequately support the health of pregnant Canadian women and to ensure the most effective strategies of public awareness, screening and intervention, it is important to understand the varied characteristics of the women undertaking these behaviours. Older maternal age is reported frequently as robustly associated with alcohol consumption during pregnancy [24,27-29]. Cigarette smoking during pregnancy and use of illicit drugs such as cocaine and marijuana are also important predictors of alcohol consumption during pregnancy [1,28-31]. Pregnancy unwantedness [29], domestic violence [27,29,32], earlier gestational stage [1,3,28,33], Aboriginal status [6,25], single marital status [1,3,29], primiparity [30], low socio-economic status [30,31], pre-pregnancy drinking [24,34], and being employed [3,24] are also all reported in the literature as predictors of any alcohol consumption during pregnancy.

It appears that maternal alcohol consumption during pregnancy may potentially constitute a major public health concern in Canada. Despite this, the available epidemiological data on both rates and predictors of alcohol consumption during pregnancy is lacking. There are few recently published peer-reviewed Canadian studies. There also exists a scarcity in regionally variable data, with a disproportionate focus on small and generally rural sub-populations, which may lead to a consensus which is not generalizable to the Canadian population on a whole. Other studies have focused on specific patterns of alcohol consumption or clinical diagnoses of Fetal Alcohol Syndrome. Finally, very few data exist for the various correlates or risk markers for prenatal alcohol use [35], especially within Canada [36]. The objective of the present study was to assess the prevalence and predictors of maternal alcohol consumption during gestation among Canadian women. Prevalence rates were assessed separately for the Eastern-Central provinces, Western Provinces-British Columbia, Eastern-Atlantic provinces and Western-Prairie Provinces.

## Methods

Data from the Maternity Experience Survey (MES) was analyzed in this study. The MES was sponsored by the Public Health Agency of Canada (MES Group of the Canadian Perinatal Surveillance System) and conducted by Statistics Canada in 2006. The MES study is a nationwide survey that assessed pregnancy, delivery and postnatal experiences of mothers and their children. Participants eligible for the study were women aged 15 years and above, who had singleton live births between the period of February 15, 2006 and May, 2006 in the provinces of Canada and between November 1, 2005 and February 1, 2006 in the territories of Canada and who lived with their baby at the time of data collection. A stratified random sample of 8,542 Canadian women was selected without replacement from the 2006 Canadian Census of Population. Around 8,244 women were estimated to have met the eligibility criteria of the study. A total of 6,421 women, however, responded to the survey. Non-response to the survey was mainly from inability to establish contact with the mothers. Prior to data collection, an introductory letter and survey pamphlet were mailed to the women and invited them to participate in the survey. Then the data was collected through telephone interviews using a computer-assisted telephone interview application. In an attempt to recruit the highest number of mothers possible, a total of 25 calls per case were made during different days of the week and different hours of the day. The MES questionnaire was also available in 15 languages. Majority of the interviews were conducted between the 5^th ^and 9^th ^month after delivery and lasted on average 45 minutes. The MES project was presented to Health Canada's Science Advisory Board, Health Canada's Research Ethics Board and the Federal Privacy Commissioner and was approved by Statistics Canada's Policy Committee. Statistics Canada's data integrity and confidentiality guidelines were strictly adhered to throughout this study. The MES has been previously described in other references [37]. For the purposes of this study, data from the Yukon, Northern Territories, Nunavut and Aboriginal individuals were excluded.

The main outcome variable assessed was ever drinking alcohol during pregnancy. This variable was derived from the question "After you realized you were pregnant, how often did you drink alcoholic beverages?" and categories included: everyday, 4-6 times per week, 2-3 times per week, once per week, 2-3 times per month, once per month, less than once per month or never. Those who reported never drinking during pregnancy were grouped as never drinkers and those who reported all other frequencies of drinking were grouped as ever drinkers. 95.8% of the ever drinker sample consumed alcohol at low to moderate levels (≤1 drink per day). Therefore, the prevalence and predictors identified in our analysis mainly reflect that of low to moderate maternal alcohol consumption. Potential predictors investigated included: i) socio-economic factors: maternal years of education, total household income, maternal work status during pregnancy and place of residence; ii) demographic factors: immigration status and province of residence; iii) maternal characteristics: marital status, age at first pregnancy, parity, age at selected birth, and mother's perceived health; and iv) pregnancy related factors: self reported weight gain during pregnancy, smoking during pregnancy, support during pregnancy, mother's reaction to pregnancy, mother's stress level before and during pregnancy, health problems during pregnancy, attendance of prenatal classes and number of prenatal care visits. All variables, except for mother's stress level, were assessed directly through specific questions. Mother's stress level was measured through a set of 13 'yes or no' questions that examined the mother's experience of stressful events in the 12 months before the birth of her selected child. The sum of the 'yes' responses were calculated to represent total stress level.

The prevalence of drinking was estimated through population weights and examined across the Canadian provinces and territories. Applying the appropriate sample weights to the MES data allowed the survey data to be representative of the population. Please refer to Statistics Canada's *Maternity Experiences Survey, 2006 - User Guide *for further information:

<http://www.statcan.gc.ca/imdb-bmdi/document/5019_D1_T1_V1-eng.pdf>.

At the bivariate level, differences in the proportion of drinkers were assessed among the different levels of each predictor using normalized weights. Odds Ratios (OR) using 95% Confidence Intervals (95% CI) were performed for categorical variables. Means and standard deviations were reported for continuous variables. Factors that proved to be significant at the bivariate level were considered for a multivariable logistic regression analysis. Adjusted OR and 95% CIs were reported in the final model. To account for the complex sampling design of the MES, bootstrapping was performed to calculate all the 95% CI estimates. All analyses, in exception to bootstrapping, were conducted using SPSS, Version 16.0. Bootstrapping was performed using SAS, Version 9.2.

## Results

**Figure 1:**
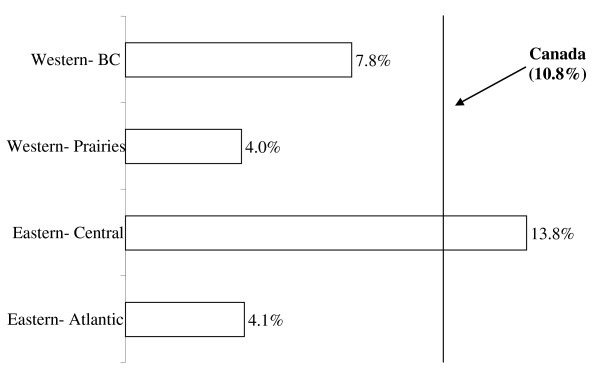
**Distribution of alcohol drinking during pregnancy across the Canadian provinces (2005/06)**. Eastern Atlantic: Newfoundland & Labrador, Nova Scotia, Prince Edward Island & New Brunswick; Eastern Central: Quebec & Ontario; Western Prairies: Manitoba, Saskatchewan, & Alberta; Western British Columbia: British Columbia; Northern Territories: Yukon Territory, Nunavut & Northwest Territories.

The sample size for the population analyzed in this study, after excluding Aboriginal individuals as well as residents of The Yukon, The Northern Territories and Nunavut was 5882, weighted to represent 72,767. Aboriginals constituted 4.2% of the total MES population, while residents of the Northern Territories and the Yukon constituted 0.5%. As shown in Table 1, a total of 45,260 (62.3%) Canadian women reported drinking alcohol before their pregnancies. 64,728 (89.2%) of women reported never drinking alcohol during their pregnancies, while 7799 (10.8%) reported ever drinking alcohol during their pregnancy. As illustrated in Figure 1, 13.8% of women in Eastern-Central provinces reported using alcohol during pregnancy, while women in Western Provinces-British Columbia, Eastern-Atlantic provinces and Western-Prairie provinces reported prevalence rates of 7.8%, 4.1% and 4.0%, respectively. These prevalence rates mainly reflect low to moderate levels of maternal alcohol consumption.

**Table 1:** Distribution of smoking before and during pregnancy among Canadian mothers (2005/06)

	N*	% (95% CI)†
Drinking alcohol before pregnancy	45,260	62.3 (61.0, 63.5)
Drinking alcohol during pregnancy	7,799	10.8 (10.0, 11.5)
Drinking alcohol during pregnancy frequency		
Never	64,728	89.2 (88.5, 90.0)
Less than once a month	5,488	7.6 (6.9, 8.3)
Once a month	1,353	1.9 (1.5, 2.2)
2 to 3 times a month	393	0.5 (0.4, 0.7)
Once a week	474	0.7 (0.4, 0.9)
2 to 3 times a week	91	0.1 (0.0, 0.2)
4 to 6 times a week	0	-
Everyday	0	-
Number of drinks per day during pregnancy‡		
Less than 1 drink	3,320	42.6 (38.5, 46.8)
1 drink	4,138	53.2 (49.0, 57.3)
2 drinks	196	2.5 (1.2, 3.8)
3 or more drinks	130	1.7 (0.6, 2.7)

*Sample size is estimated using population weights

† 95% CIs were calculated using bootstrapping technique

‡ The total sample for this variable constitutes only those who drink alcohol during pregnancy

Table 2 reports unadjusted Odds Ratios and 95% Confidence Intervals performed on alcohol utilization during pregnancy and all other socio-economic, demographic, maternal and pregnancy-related variables. Also reported are the results of a logistic regression analysis, where the dependent variable is alcohol utilization during pregnancy and independent variables include all socio-economic, demographic, maternal and pregnancy-related variables which were found to be statistically significant at an α of 0.05 at the bivariate level.

**Table 2:** Unadjusted and adjusted associations between alcohol drinking during pregnancy and potential predictors

	Sample Size	Alcohol Drinking During Pregnancy	Unadjusted Odds Ratio	Adjusted Odds Ratio
	N*	N (%)	OR (95% CI)†	OR (95% CI)†
Household income (Canadian dollars)				
<$30,000	925	77 (8.3)	1	1
$30,000 to less than $60,000	1,771	173 (9.8)	1.20 (0.89, 1.61)	0.90 (0.63,1.28)
$60,000 to less than $100,000	1,882	241 (12.8)	**1.62 (1.23, 2.14)**	1.08 (0.75, 1.56)
≥$100,000	1,180	151 (12.8)	**1.62 (1.19, 2.20)**	0.94 (0.61, 1.44)
Place of residence				
Rural area	1,034	138 (13.3)	1	1
Urban, population ≤499,999	2,130	196 (9.2)	0.66 (0.51, 0.84)	0.77 (0.59, 1.00)
Urban, population ≥500,000	2,713	307 (11.3)	0.83 (0.66, 1.04)	0.95 (0.73, 1.23)
Immigrant				
No	4,673	566 (12.1)	**2.09 (1.60, 2.71)**	**2.49 (1.77, 3.48)**
Yes	1,402	87 (6.2)	1	1
Work during pregnancy				
No	1,259	103 (8.2)	1	1
Yes	4,822	551 (11.4)	**1.44 (1.14, 1.84)**	1.28 (0.95, 1.71)
Marital status				
No partner	462	37 (8.0)	1	1
Have a partner	5,624	618 (11.0)	1.43 (1.00, 2.03)	**2.00 (1.20, 3.31)**
Moms perceived health				
Excellent/very good	4,448	489 (11.0)	1	--
Good	1,333	141 (10.6)	0.96 (0.77, 1.19)	--
Poor/Fair	304	25 (8.2)	0.71 (0.44, 1.15)	--
Smoking during pregnancy				
No	5,499	570 (10.4)	1	1
Yes	588	84 (14.3)	**1.45 (1.12, 1.87)**	**1.54 (1.10, 4.14)**
Health problems during pregnancy				
No	4,603	508 (11.0)	1	--
Yes	1,478	146 (9.9)	0.88 (0.72, 1.08)	--
Reaction when discovered pregnancy				
Very happy/happy	5,660	586 (10.4)	1	1
Indifferent	242	35 (14.5)	1.47 (1.00, 2.17)	**1.89 (1.21, 2.94)**
Very unhappy/Unhappy	163	28 (17.2)	**1.77 (1.13, 2.79)**	**2.50 (1.47, 4.24)**
Attended prenatal classes				
No	4,095	442 (10.8)	1.02 (0.84, 1.23)	--
Yes	1,990	211 (10.6)	1	--
Support during pregnancy				
None/Little of time	313	39 (12.5)	1.24 (0.86, 1.79)	1.13 (0.72, 1.77)
Some of the time	478	66 (13.8)	**1.38 (1.02, 1.85)**	1.37 (0.97, 1.93)
Most/All of time	5276	547 (10.4)	1	1
Province‡				
Eastern-Atlantic	365	15 (4.1)	1	1
Eastern-Central	3,918	541 (13.8)	**3.72 (2.77, 4.99)**	**3.89 (2.80, 5.39)**
Western-Prairies	1,101	44 (4.0)	0.98 (0.65, 1.45)	0.94 (0.61, 1.46)
Western-British Columbia	704	55 (7.8)	**1.95 (1.27, 3.00)**	**2.14 (1.35, 3.40)**
		**Alcohol Drinking During Pregnancy**	**Non-Alcohol drinking during pregnancy**	
		**Mean (SD)**	**Mean (SD)**	
Years of education	6,046	15.49 (3.24)	14.91 (2.94)	1.04 (1.00, 1.09)
Age at first pregnancy	6,017	25.83 (5.61)	25.45 (5.23)	--
Number of past pregnancies	6,080	2.55 (1.71)	2.26 (1.38)	**1.09 (1.02, 1.17)**
Mother's age at selected birth	6,051	30.90 (4.98)	29.64 (5.16)	**1.04 (1.02, 1.07)**
Weight gained during pregnancy	6,019	15.49 (6.07)	15.63 (7.54)	--
Number of stressful events	6,040	1.33 (1.49)	1.22 (1.46)	--
Number of prenatal visits	5,838	12.33 (4.56)	13.00 (4.68)	**0.97 (0.94, 0.99)**

* Sample size is estimated using normalized weights.

† 95% CI were calculated using bootstrapping technique.

‡ Eastern Atlantic: Newfoundland & Labrador, Nova Scotia, Prince Edward Island & New Brunswick; Eastern Central: Quebec & Ontario; Western Prairies: Manitoba, Saskatchewan, & Alberta; Western British Columbia: British Columbia; and Northern Territories: Yukon Territory, Nunavut & Northwest Territories.

Socio-economic variables considered in the analysis included maternal years of education, total household income, maternal work status during pregnancy and place of residence. While prior to adjustment, all of these factors appeared to be significant to some effect, none of these relationships persisted in the adjusted model. Demographic variables considered in the analysis included immigration status and province of residence. Being an immigrant appears to confer a protective effect, as immigrants were less likely to use alcohol during pregnancy than native-born women (OR = 2.49; 95% CI: 1.77, 3.48). Residing in Eastern-Central provinces (OR = 3.89; 95% CI: 2.80, 5.39) and Western Provinces-British Columbia (OR = 2.14; 95% CI: 1.35, 3.40) as compared with Eastern-Atlantic provinces also remained significant prior to adjustment. Maternal characteristics considered in the analysis included marital status, age at first pregnancy, parity, age at selected birth and mother's perceived health. Following adjustment, the analysis demonstrated that women who have marital partners are twice as likely to drink during pregnancy than women without partners (OR = 2.00; 95% CI: 1.20, 3.31). Age at selected birth also remained significant (OR = 1.04; 95% CI: 1.02, 1.07), as did number of past pregnancies (OR = 1.09; 95% CI: 1.02, 1.17). Pregnancy-related factors considered in the analysis include self-reported weight gain during pregnancy, smoking during pregnancy, support during pregnancy, mother's reaction to pregnancy, mother's stress level before and during pregnancy, health problems during pregnancy, attendance of prenatal classes and number of prenatal care visits. Smoking during pregnancy remained significant and was associated with increased likelihood of drinking during pregnancy (OR = 1.54; 95% CI: 1.10, 4.14). Mother's reaction to pregnancy proved to have a significant association, with those women who reported being indifferent to their pregnancies drinking during pregnancy 1.89 times more than women who reported being very happy or happy (95% CI: 1.21, 2.94) and women who reported being very unhappy or unhappy drinking during pregnancy 2.50 times more than women who reported being very happy or happy (95% CI: 1.47, 4.24). Number of prenatal care visits remained significant following adjustment, though a much weaker association was observed (OR = 0.97; 95% CI: 0.94, 9.99).

## Discussion

The present study assessed the prevalence of maternal alcohol consumption during pregnancy, as well as predictors of this behavior among Canadian women. The analysis mainly focused on low to moderate levels of alcohol consumption (≤1 drink per day) since this constituted the majority of our sample of mothers who consumed alcohol during pregnancy. Results indicated that alcohol utilization during pregnancy continues to constitute a major Canadian health problem, with over 10% of women continuing to use alcohol while pregnant, despite widespread knowledge regarding the adverse effects and the understanding that no amount of alcohol ingestion during the gestational period can be deemed safe with the current body of inconclusive evidence surrounding fetal damage across all levels of alcohol consumption, especially low to moderate levels of drinking. Women who are residents of Eastern Central or Western provinces, women with partners, women who smoke and women who have indifferent or unhappy reaction to their pregnancies had increased likelihood of using alcohol during their pregnancy. Alternatively, being an immigrant appeared to have a protective effect, with women who were immigrants demonstrating decreased likelihood of using alcohol during their pregnancies.

In the present study, drinking alcohol during pregnancy was reported to be 10.8%. This rate is comparable to the rates reported in similar studies within Canada and the United States, as well as by Health Canada [20,21,23,24,27,28,38-40]. Survey data from 1994-1995 reports 17-25% of pregnant women consuming any alcohol while pregnant, which decreased to 7-9% in 1998-1999, though one 1998-1999 National Longitudinal Survey of Children and Youth (NLSCY) reported 14.1% of women consuming alcohol during pregnancy [10].

At the multivariable level, none of the socio-economic variables, including maternal years of education, total household income, maternal work status during pregnancy and place of residence were found to be significant. Findings in the current literature however are inconclusive, with some studies reporting an association between higher levels of socio-economic variables and alcohol use during pregnancy [24,38,41,42]. Alternatively, other studies have found that lower levels of socio-economic variables are associated with prenatal exposure to alcohol [43,44]. Still other studies have shown no association, similar to the results of the present study [28,45]. These disparities may be due to differences in the variables controlled at the analytic level.

With regard to demographic variables, both immigrant status and province of residence were demonstrated as significant following multivariable analysis. These findings are consistent with other studies which have indicated that immigrant women and women of certain cultural groups are less likely to use alcohol during pregnancy [1,24,27,29,39,41]. This may be due to differing social, behavioural and religious norms and beliefs or support networks experienced by women of certain cultural backgrounds; however, this clearly varies by cultural group and warrants further investigation. The results indicated that women in Eastern-Central and Western Provinces-British Columbia were at increased risk of drinking alcohol during pregnancy as compared with Eastern-Atlantic Provinces. Studies reporting regionally variable data including all of the Canadian provinces and territories were difficult to locate, however data reported from the 1998-1999 NLSCY indicated that the highest rates of alcohol use by pregnant women were in Quebec, and the lowest rates were found in Atlantic Canada, in agreement with the results of the present study [20]. Analysis of the 2007-2008 Canadian Community Health Survey (CCHS) data reported Ontario, British Columbia, and nationwide rates at 5.4%, 7.2%, and 5.8%, respectively [46]. This present study corroborates the BC findings; however our Eastern-Central and Canada-wide estimates are higher. Interestingly, at the multivariable level we found that women with partners were twice as likely to use alcohol during pregnancy as their single counterparts (OR = 2.00; 95% CI: 1.20, 3.31). While this is contradictory to what has been found in other similar studies [27,31,36,46], some studies have indicated that paternal or spousal alcohol utilization is associated with maternal alcohol utilization during pregnancy [43,44]. However, spousal alcohol utilization was not measured in the present study and therefore it is impossible to understand if the results are a function of this relationship. This finding however, is worthy of further investigation in future research. In regards to maternal age, results are conflicting in the current literature, with some studies indicating that younger women are more likely to drink during pregnancy [1,39,43] while others indicate that older women are more likely to use alcohol while pregnant [10,24,27,28,41,44,45]. This may be due to the varying patterns observed of alcohol utilization, such as younger women being more likely to engage in binge drinking than their older counterparts, while older women are more likely to report any drinking during pregnancy [41]. With regards to pregnancy-related variables, smoking during pregnancy, reaction to pregnancy and number of prenatal visits proved significantly associated with drinking during pregnancy. Results indicated that smoking during pregnancy was associated with greater likelihood of drinking alcohol during pregnancy (OR = 1.54; CI: 1.12, 1.87). This finding is confirmed by previous literature [3,28,30,41,43]. Canadian studies evaluating maternal smoking cessation programs are sparse, and the results are inconsistent. Some studies report the ineffectiveness of intervention [47], while others report that cessation rates were 2-3 times higher in the intervention group [48]. In the adjusted model, number of prenatal visits conferred a very modest protective effect (OR = 0.97; CI: 0.94, 0.99), however; our unadjusted results (crude OR = -0.66; CI : -1.06, -0.27) corroborate unadjusted healthcare utilization findings looking at the effect of visiting a general practitioner in the past year (crude OR = 0.520; CI 0.30, 0.92) [42]. The present study revealed that reaction to pregnancy was one of the strongest correlates of alcohol use during pregnancy, with those who were indifferent having close to double the risk of drinking during pregnancy than those who were happy (OR = 1.89; 95% CI: 1.21, 2.94) and those who were very unhappy or unhappy having two and a half times more risk (OR = 2.50; 95% CI: 1.47, 4.24). This is also supported elsewhere in the literature [27,41].

The present study has several strengths. Firstly, the present study considered data from all Canadian provinces, resulting in a representative picture of the population, along with the ability for provincial comparison. The present study also had a high response rate of 78%, but as is the case with all survey studies, selection bias may be a factor. This study also utilized a large sample size, allowing for ample statistical power. The population weights, also, created by Statistics Canada and used in the analysis accounted for the non-response. Additionally, a wide variety of potential predictors were considered and controlled for within the analysis, conferring greater likelihood that confounding factors were minimized. One possible limitation of the present study was its reliance on self-report measures to determine the presence and degree of exposure to alcohol during pregnancy. Recall bias may have been a factor since interviews were conducted 5-9 months post-delivery. A further limitation is that causality cannot be inferred because of the cross-sectional nature of the data. As reported by previous studies, the rates of maternal alcohol consumption may be underestimated due to the sensitive and stigmatized nature of the behavior being measured [49]. Our results could have been more informative if we grouped subjects into low, moderate, and heavy drinkers rather than dichotomizing the outcome. However, the small cell sizes that resulted after this categorization did not meet MES confidentiality guidelines. Moreover, without information on peak blood alcohol level and timing of alcohol drinking [50], maternal alcohol consumption does not provide enough information to determine fetal alcohol exposure. Additionally, MES data on alcohol utilization among Aboriginal populations reported extremely low prevalence of alcohol drinking during pregnancy, while the previous literature has demonstrated that Canadian Aboriginal populations have higher rates of alcohol utilization during pregnancy [6,10,25] and therefore data collected from these populations was excluded for the purposes of this study in an attempt to reduce potential reporting bias. This may slightly limit the generalizability, as the rates presented in this study may not be representative of these Canadian subpopulations. The reason for the regional differences in alcohol consumption during pregnancy is not clear. It can be speculated to differences in culture, antenatal care, or immigration status. Further qualitative studies should look into these differences. In addition, multilevel analysis using register-based data linked to nationwide survey data, aiming to disentangle individual versus regional area level effects might add further insight to this area.

## Conclusions

This study has identified an important public health priority since fetal alcohol exposure is associated with birth defects and a massive financial burden on the economy. Such interventions are critical to meeting goals for guiding primary and secondary prevention efforts, informing effective public health research and promoting maternal and fetal health, along with the health of the Canadian population. While a vast majority of women greatly reduce or completely abstain from alcohol use during pregnancy, some continue to use alcohol. In Canada, around ten percent of women continue to drink alcohol during their pregnancies, a period critically important in terms of embryonic and fetal development. We recommend future research look into the effects of low to moderate maternal alcohol consumption in comparison to heavy and binge drinking.

These results have attempted to identify the relevant risk factors for prenatal alcohol utilization, providing justification for increased development and implementation of interventions among pregnant women. It appears that non-immigrant mothers should receive an increased focus. Additionally, it appears that women residing in the Eastern-Central and Western provinces require special attention, as province of residence emerged as the strongest associated variable in the present study. Finally, increased interventions to decrease unplanned pregnancy combined with increased support interventions for women experiencing unwanted pregnancies appear critical for reducing prenatal alcohol use.

## Abbreviations

CCHS: Canadian Community Health Survey; CI: Confidence interval; FAE: Fetal Alcohol Effects; FAS: Fetal Alcohol Syndrome; MES: Maternity Experience Survey; NLSCY: National Longitudinal Survey of Children and Youth; OR: Odds Ratio; SAS: Statistical Analysis Software; SD: Standard deviation; SPSS: Statistical Package for Social Sciences.

## Competing interests

The authors declare that they have no competing interests.

## Authors' contributions

MJW performed the literature review and the write of the paper. BAS performed the analysis and contributed in writing the manuscript. FI addressed reviewers' concerns and contributed to the writing and editing of the manuscript. HT generated the idea of the research and supervised the analysis and write up of the manuscript.

All authors read and approved the final manuscript.

## Pre-publication history

The pre-publication history for this paper can be accessed here:

http://www.biomedcentral.com/1471-2393/11/52/prepub
